# Treatment of rabbit cheyletiellosis with selamectin or ivermectin: a retrospective case study

**DOI:** 10.1186/1751-0147-50-1

**Published:** 2008-01-02

**Authors:** Marianne Mellgren, Kerstin Bergvall

**Affiliations:** 1 Djurklinik Stigsbo, Stigsbo 213, Stjärnsund, Sweden; 2 Department of Small Animal Clinical Sciences, Faculty of Veterinary Medicine and Animal Science, Swedish University of Agricultural Sciences, Uppsala, Sweden

## Abstract

**Background:**

A retrospective study of rabbits treated against cheyletiellosis was performed to evaluate the efficacy and safety of selamectin or ivermectin in clinical practice.

**Methods:**

Medical records from 53 rabbits with microscopically confirmed *Cheyletiella *infestation were collected from two small animal clinics. The rabbits were divided into three groups, based on treatment protocols. Group 1 included 11 rabbits treated with ivermectin injections at 200–476 μg kg^-1 ^subcutaneously 2–3 times, with a mean interval of 11 days. In Group 2, 27 rabbits were treated with a combination of subcutaneous ivermectin injections (range 618–2185 μgkg^-1^) and oral ivermectin (range 616–2732 μgkg^-1^) administered by the owners, 3–6 times at 10 days interval. The last group (Group 3) included 15 rabbits treated with selamectin spot-on applications of 6.2–20,0 mgkg^-1^, 1–3 times with an interval of 2–4 weeks. Follow-up time was 4 months–4.5 years.

**Results:**

Rabbits in remission were 9/11 (81,8%), 14/27 (51,9%) and 12/15 (80,8%) in groups 1, 2 and 3, respectively.

**Conclusion:**

All treatment protocols seemed to be sufficiently effective and safe for practice use. Though very high doses were used in Group 2 (ivermectin injections followed by oral administration), the protocol seemed less efficacious compared to ivermectin injections (Group 1) and selamectin spot on (Group 3), respectively, although not statistically significant. Controlled prospective studies including larger groups are needed to further evaluate efficacy of the treatment protocols.

## Background

Parasite infestation with *Cheyletiella *species is reported worldwide. The condition is highly contagious, with three species, *C. parasitivorax, C. blakei *and *C. yasguri *normally infesting rabbits, cats and dogs respectively [1-3]. *Cheyletiella *mites belong to the order *prostigmata *and the family *Cheyletiellidae*. The mites are large (270–540 μm) and very mobile. Adult mites have four pairs of legs with distal combs instead of claws. The mouthpart (palpi) has a pair of curved claws characteristic of the mite *Cheyletiella*. The three species can be separated morphologically only by the shape of a sensory organ in genu 1 on the first pair of legs. The entire lifecycle of approximately 21 days is spent on the host, where they feed on surface epithelia, debris and lymph. Adult *Cheyletiella *mites can survive a month without feeding in cool temperatures [2,3]. Eggs that fall off the host can be a source of re-infestation.

**Figure 1:**
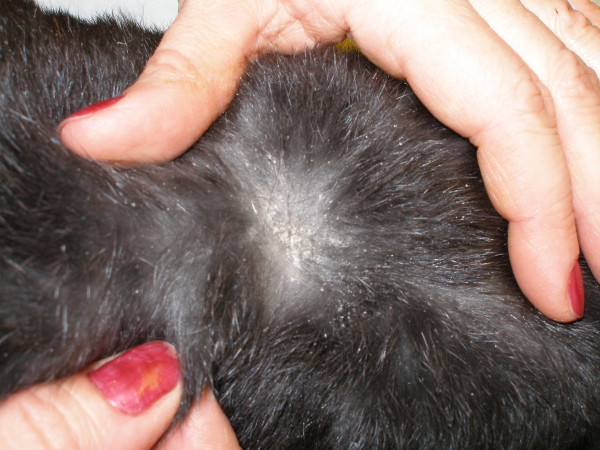
**Rabbit with cheyletiellosis**. Rabbit with cheyletiellosis, with typical signs of seborrhoea and scaling over the withers and back.

Cheyletiellosis is a zoonosis, causing papular, pruritic dermatitis in humans [1,2]. Experimental studies transferring *C. yasguri *between dogs and rabbits suggest a low host specificity of the mite [1]. Cheyletiellosis in the rabbit is common and clinical signs consist of varying degrees of pruritus, alopecia, dry to oily seborrhoea with crusts and sometimes erythema (figure 1) [1]. Lesions mainly affect the withers but sometimes extend to the back and ventral abdomen. In some cases lesions are seen only in the face. The severity of clinical signs ranges from asymptomatic to moderate, although widespread lesions can be seen in immunocompromised rabbits [1-3].

Diagnosis of *Cheyletiella *infestation is made by demonstration of the mite or eggs in superficial skin scrapings, transparent tape preparations or by flea combing.

A variety of antiparasitic treatment protocols have been successfully used for ectoparasitic mite infestation in small animal veterinary practice. In the literature there is evidence of efficacy using fipronil [3,4], ivermectin [5-8] and selamectin [9,10] in the treatment of cheyletiellosis in the dog and cat. Use of ectoparasiticides in the rabbit has been reported in the treatment of *Psoroptes cuniculi *and *Sarcoptes scabei *with ivermectin [11-13], ivermectin and fipronil combination [14], moxidectin [15], eprinomectin [16], selamectin [17,18] and doramectin [19]. Although ivermectin, selamectin and moxidectin have been advocated for treatment of cheyletiellosis in rabbits in textbooks and proceedings [2,20,21], no studies to our knowledge, have been published to support the recommendation. In the view of evidence-based medicine, it is important that primary references are available to substantiate textbook statement and clinical practice. The objective of the present study was therefore to evaluate the efficacy and safety of selamectin and ivermectin in the treatment of *Cheyletiella *mite infestation in rabbits in clinical practice.

## Methods

### Study design and inclusion criteria

Totally 282 medical records of rabbits from two veterinary settings (Gästrike Animal Clinic, Sandviken, Sweden, and Animal Clinic Roslagstull, Stockholm, Sweden) were reviewed. Of these, 53 rabbits were included in the study. Prerequisites for selection were that clinical signs compatible with *Cheyletiella *infestation were verified by a demonstration of *Cheyletiella *mites through light microscopy examination of skin scrapings, material from flea combing or transparent tape preparations under 4 or 10 × 100 magnifications. Furthermore, the rabbits should have been treated with ivermectin or selamectin and a follow-up should be possible through telephone contact with the owner or through a revisit at the clinic. The following information was collected from the two veterinary settings: descriptions including age, sex, weight, clinical signs, diagnose verification, treatment protocol (substance, dose, route of administration and interval), number of rabbits in the household, other treatments and evidence of concurrent diseases. A record of overweight was made if the rabbit's weight was more than 20% above the recommended maximum weight of the breed according to the Nordic rabbit standard [22].

The rabbits were divided into three treatment groups, ivermectin (Ivomec^®^vet. injectable, 10 mg/ml, Merial SAS, Lyon) injections (Group 1), combination of injections and oral administration of ivermectin (Ivomec^®^vet. injectable, 10 mg/ml, Merial SAS, Lyon) (Group 2) and topical selamectin (Stronghold^®^/Revolution, 60 mg/ml, Pfizer Inc., New York) (Group 3).

### Treatment groups

Group 1 included 11 rabbits, all treated at the Gästrike Animal Clinic, with a mean age of 4.4 years (range 9 months to 7 years) with bodyweights ranging from 1.4 to 4.6 kg. Eight rabbits were male and three female. Most of the rabbits (n = 9) were from single-rabbit households. In the two multi-rabbit households, all in-contact rabbits were treated. The rabbits were treated with ivermectin injections subcutaneously at two (n = 5) or three (n = 6) occasions. The mean dose was 253 μg kg^-1 ^(range 200–476) and the mean injection interval was 11 days (range 9–21).

Group 2 included 27 rabbits, all treated at the Animal Clinic Roslagstull, with bodyweights ranging from 1.6 to 6.5 kg and with a mean age of 4 years (range 6 months to 9.5 years). Twenty rabbits were intact males while the rest were four females and three castrated males. Most rabbits lived in single-rabbit households (n = 24) in this group as well, and the rest in households with two or more rabbits. All in-contact rabbits were treated in the multi-rabbit households. Treatment consisted of 3–6 ivermectin administrations at a 10 day interval. Initial subcutaneous injections at the first visit (mean dose of 1044 μgkg^-1^, range 618 to 2185) were followed by oral ivermectin, using the injectable formula (mean dose of 1324 μgkg^-1^, range 616–2732) twice and administered by the owner. Most rabbits (n = 23) were re-examined 30 days (range 28–35 days, one after 48 days) after the first visit. Depending on the clinical signs at the second visit they were either given no more treatment (n = 2) or continued treatment. Twenty-one rabbits had a second injection, 14 of which had two additional oral ivermectin treatments repeated, with the same doses and interval as initially. Most of the latter were considered to still have clinical signs (n = 8) or to be mite positive after microscopic control (n = 1).

Group 3 included 15 rabbits: one treated at the Gästrike Animal Clinic and 14 at the Animal Clinic Roslagstull. The mean age in this group was 2.2 years (range 3 months to 7 years) with bodyweights ranging from 1 to 7.4 kg. Eight rabbits were intact males while the rest were 5 females and 2 castrated males. Two rabbits belonged to multi-rabbit households. All in-contact rabbits were treated. Treatment consisted of administration of selamectin spot on topically at 1–3 occasions. The mean dose of selamectin used was 12.5 mgkg^-1 ^(range 6.2 to 20.0).

### Assessment of response to therapy

Clinical cure was assessed by re-examination or telephone contact with the owner. Treatment results were graded as in remission, relapse or treatment failure at the time of follow-up. Rabbits in remission were defined as having been free from clinical signs at re-examinations during the whole follow-up period. Relapses were defined as being free from clinical signs more than 3.5 months after treatment but showing signs again during the follow-up time. Treatment failures were cases that never cleared from clinical signs during the first 3.5 months or were recorded with relapse during this time. Adverse reactions of treatment were assessed by examination during revisit and by questioning the owner.

Statistical analysis was made by using χ^2 ^test [23].

## Results

### Group 1: Ivermectin injections

Nine rabbits out of 11 were classified as in remission at follow-up, whereas one was graded as treatment failure and one was recorded as relapse (table 1). Most of the rabbits graded as in remission had no clinical signs (n = 5) or were mite negative (n = 1) as assessed by the veterinarian at revisit. The remaining three rabbits were reported free from clinical signs by telephone contact with the owner. The rabbit considered as a treatment failure was placed in a new cage during treatments. This rabbit had concurrent signs of conjunctivitis and dental problems; it died two months after the last treatment. One rabbit experienced a microscopically confirmed relapse two years after treatment and was re-treated. This rabbit had concurrent back pain. Shampoo containing bioallethrin and piperonylbutoxid (Dermocan, Dogman) was advocated at the first occasion. In the remission group, three rabbits were recorded as having concurrent diseases (conjunctivitis, dental problems and back pain) and three were recorded as overweight. Four rabbits were concurrently bathed with sulphur shampoo (<5% sulphur) once or twice weekly in order to reduce the seborrhoea. Environmental treatment was conducted in three cases. One rabbit had its cage cleaned mechanically, one cage was cleaned with fipronil spray (2.5 mgml^-1^) (Frontline vet, Merial) and one with Dermocan-shampoo. Side effects (ataxia, resolving before the third injection) were reported in one rabbit. Although mite-negative at the last visit, this rabbit was found dead a few months after the last treatment. One rabbit experienced pain during injection of ivermectin. The follow-up time in this group ranged from 4 months to 3.5 years (mean time 18 months).

**Table 1:** Treatment results of rabbits treated with ivermectin injections. Treatment results of rabbits treated with ivermectin injections at 11 day (range 9–21 days) intervals. Relative outcome (%) in brackets.

No. of ivermectin injections	Treatment response (result)			Total
		
	In remission	Failure	Relapse	
Two	5 (45.5)	0	0	5 (45.5)
Three	4 (36.5)	1 (9.0)	1 (9.0)	6 (54.5)

Total	9 (82.0)	1 (9.0)	1 (9.0)	11(100)

### Group 2: Ivermectin injection followed by oral ivermectin administration

Out of 27 rabbits, 14 were considered as in remission at follow-up. Among those, nine were examined at re-visit (two were skin scraped and confirmed microscopically negative) and five were assessed through telephone contact with the owner. Overweight status was recorded in three rabbits and one rabbit had concurrent conjunctivitis. The cage of one rabbit was cleaned mechanically, confirmed by telephone contact with the owner. Relapse, microscopically verified, was seen in seven rabbits within 7 months to 2 years after the last treatment. In this group one rabbit was overweight and one was in general poor condition at relapse. Six rabbits (four confirmed by visit and two by telephone contact) were graded as treatment failures and still showed clinical signs after treatment with 3–6 ivermectin doses (table 2). One of these had concurrent conjunctivitis and dental problems and one was overweight. Follow-up time in Group 2 was 7 months to 4.5 years (mean time 13.7 months).

**Table 2:** Treatment results of rabbits treated with ivermectin injections and oral administration. Treatment results of rabbits treated with ivermectin injections and oral administration according to different protocols. Treatment interval 10 days, second injection after 30 days (range 28–35, one after 48 days). Relative outcome (%) in brackets.

Treatment protocol	Treatment response (result)			Total
		
	In remission	Failure	Relapse	
Protocol 1 1 injection + 2 oral doses	3 (11.1)	1 (3.7)	2 (7.3)	6 (22.2)
Protocol 2 1 injection, 2 oral doses followed by 1 injection	4 (14.8)	1 (3.7)	2 (7.3)	7 (25.9)
Protocol 3 1 injection, 2 oral doses, 1 injection and 2 oral doses	7 (25.9)	3 (11.1)	4 (14.8)	14 (51.4)

Total	14 (51.9)	5 (18.5)	7 (25.9)	27 (100)

### Group 3: Topical selamectin

Twelve rabbits out of 15 were assessed as in remission. Ten of the twelve were assessed through telephone contact and two through re-examination. In this group four rabbits were recorded as having other diseases (three had a stiff back and one had conjunctivitis) and three were overweight. Two rabbits had their cages cleaned mechanically. The only rabbit considered to be a treatment failure was overweight. Two rabbits relapsed within 3.5 and 8 months, respectively, after the last application and were re-treated with the same treatment protocol (table 3). One of them was in generally poor condition at relapse. Follow-up time in Group 3 was 4 months to 1 year 5 months (mean 8.2 months).

**Table 3:** Treatment results of rabbits treated with selamectin spot-on. Treatment results of rabbits treated with selamectin spot-on according to different protocols. Relative outcome (%) in brackets.

Treatment protocol		Treatment response (result)			Total
		
No. of treatments	Interval	In remission	Failure	Relapse	
1		2 (13.3)	0	0	2 (13.3)
2	1 month	6 (40.0)	1(6.6)	1 (6.6)	8 (53.3)
3	1 month	2 (13.3)	0	0	2 (13.3)
3	3 weeks	1 (6.6)	0	0	1 (6.6)
2	2 weeks	1 (6.6)	0	1 (6.6)	2 (13.3)

Total		12 (8 0.0)	1 (6.6)	2 (13.3)	15 (100)

When comparing all treatment groups with each other no significant differences were found (p = 0.09, n = 53).

## Discussion

This study indicates that all treatment protocols seemed to be sufficiently effective and safe and that cheyletiellosis in rabbits can be successfully treated using ivermectin or selamectin in clinical practice. Neither ivermectin or selamectin is currently approved for use in rabbits in Sweden. In the UK however a topical spot-on preparation containing ivermectin (Xeno 450, Genitrix) has just recently been initiated for use in rabbits, guinea pigs and ferrets.

Ivermectin and selamectin both belong to the macrocyclic lactones. All macrocyclic lactones have the same primary mode of action, namely to affect the chloride ion channel activity in the nervous system of nematodes and arthropods. Nerotoxicity however has been described in mammals, mainly in cases of ivermectin use in collies (and collie related breeds) and young animals or after overdosing [6,7].

The doses of ivermectin recommended for cheyletiellosis in dogs and cats are oral or injectable formulas at 200–300 μgkg^-1^, at a 10–14 day interval during 6–8 weeks [8]. Studies in rabbits suffering from *Psoroptes *infestation have concluded that ivermectin at the doses of 100–200 μgkg^-1 ^twice, with a 2 week interval, and of 400 μgkg^-1 ^once subcutaneously, are effective in eliminating the mite [12,13].

Selamectin has been shown to have persistent efficacy against fleas after topical administration in dogs and cats for at least 28 days after application [24]. Cheyletiellosis in a multi-cat household and in three canine breeding colonies was successfully treated with selamectin (6–15 mgkg^-1^), using 3–4 topical treatments at 2–4 week intervals [9,10]. Rabbits infested with psoroptes or sarcoptes and treated with selamectin (6–18 mgkg^-1^) spot-on applications at one or two occasions at 2–4 week intervals had a complete recovery [17,18].

The results from this field study may have been affected by uncontrolled events and factors. For example the chance that the treated rabbits in this study were asymptomatic carriers of *Cheyletiella *mites after treatment cannot be excluded, as the response to treatment was assessed as clinical cure and not parasitical cure, except in a few cases. Treatment results were therefore graded as in remission only if no relapse was seen during the entire follow-up time and as relapses if the rabbit experienced recurrence of clinical signs during the follow-up time.

There may be several explanations as to why the combination of injection and oral administration of ivermectin (Group 2) has a tendency to be less effective, independent of treatment length. It is possible for example that oral bioavailability of ivermectin is not high enough in rabbits, explaining the higher number of rabbits experiencing relapse or treatment failure. Lower efficacy and shorter duration of action from orally administered ivermectin have been documented, for instance in cats where most of the drug is eliminated from plasma at 5 days [5,7]. The doses used in Group 2 were very high but this factor does not seem to affect the treatment results, perhaps reinforcing the theory that the oral bioavailability of ivermectin is low in rabbits. In contrast subcutaneous injections of ivermectin have been shown to elicit high concentrations in plasma for at least 13 days in rabbits [13]. Another cause could be compliance-related due to owner difficulties when medicating the rabbits orally at home, or that the ivermectin was not stored under the right conditions (e.g. UV protection).

Why some rabbits still experienced clinical signs or relapsed even when living in single-rabbit households remains unanswered. Insufficient cleaning of the environment and in-contact dogs and cats not treated against *Cheyletiella *may be a source of re-infestation. Most owners were urged to clean the cages, although this cleaning could only be verified in a few cases (8/53). Five of the rabbits in Group 1 were also concurrently bathed. These treatments were made both in the treatment failure groups and in the remission group. Other diseases could possibly also influence the incidence of relapse or lack of treatment success. In 13 of the total 53 rabbits concurrent health issues were recorded and 12 were overweight. Rabbits with concurrent disease or overweight however were seen in both treatment failure and remission groups. Time to relapse varied from a few weeks to several years. Five of the rabbits with relapse were living in single-rabbit households. The mean follow-up time in Group 3 was shorter (8.2 months) compared to the two ivermectin groups (13.7–18.0 months). A study with a longer follow-up time in all groups with clinical and microscopically examination at revisit is needed to further validate the results of this study. Such a study should also include a control group. The outcome of larger groups in such a study might result in statistical significance between the different treatment groups.

Possible side effects were only recorded in two cases, both in Group 1, the group receiving ivermectin injections. One rabbit experienced pain after the ivermectin injection and one developed ataxia at the second treatment. It could not be excluded that the symptoms were correlated to the ivermectin injection. The rabbit experiencing a short episode of ataxia at the second injection died a few months after the last treatment, without having had any relapses of neurological signs. No autopsy was performed. The almost total absence of neurotoxicity is remarkable, since the doses used in Group 2 (ivermectin injection and oral administration) were very high, up to 2732 μgkg^-1^, which is many times the dosage recommended by the manufacturer for treating ruminants and horses. In ruminants the toxicity dosage (4 mg kg^-1^) is 20 times the recommended dosage, given as injections, and in horses the toxicity dosage (2 mg kg ^-1^, given twice in two days, orally) is 10 times the recommended dosage. Teratogenic studies in rabbits performed by the manufacturers concluded that ivermectin orally administrated daily to pregnant rabbits at the dose 6 mg kg^-1 ^resulted in signs of toxicity after 7 days.

## Conclusion

Results of this retrospective study suggest that both ivermectin and selamectin are effective and safe for clearance of clinical signs of cheyletiellosis in rabbits. In the group including oral administration of ivermectin (Group 2) a tendency towards a lower efficacy was registered, as compared to Groups 1 (ivermectin injections) and 3 (selamectin spot-on), although not statistically significant. Controlled prospective studies including larger groups are needed to further evaluate efficacy of the treatment protocols.

## Competing interests

The author(s) declare that they have no competing interests.

## Authors' contributions

MM collected and analyzed the data and drafted the manuscript. KB participated in planning the study and helped to draft the manuscript. All authors read and approved the final manuscript.
